# Altered cancer cell metabolism and cachexia: Calculating the energetic cost of cancer

**DOI:** 10.1186/2049-3002-2-S1-P18

**Published:** 2014-05-28

**Authors:** Douglas Friesen, Vickie Baracos, Jack Tuszynski

**Affiliations:** 1 Department of Oncology, University of Alberta, Edmonton, Alberta, Canada; 2 Department of Physics, University of Alberta, Edmonton, Alberta, Canada

## Background

Cachexia affects most patients with incurable cancer impacting quality of life and prognosis especially in the late stage of disease. While much research has investigated the causes of cancer cachexia, the precise mechanisms causing cachexia are still poorly understood. Concurrently, it is increasingly documented that tumors function with elevated glycolysis.

## Methods and Results

We model an anaerobic component of tumor energy metabolism to assess its impact and contribution to cachexia. In this model, with a high level of anaerobic energy production, the energetic cost to sustain the tumor may reach or exceed 394 kcal/ day per kg of tumor. In addition, the tumor’s high level of glucose and glutamine consumption causes muscle breakdown to fuel the tumor, especially in the fasting state. We calculate an estimate of the tumor’s energetic cost on the body in terms of aerobic and anaerobic components, as well as the Cori cycling cost of recycling lactate generated by the tumor back into glucose, at varying levels of tumor mass and of anaerobic energy metabolism.

## Conclusions

Our model suggests the energetic drain caused by a tumor is substantial when anaerobic energy metabolism is taken into account, and that elevated anaerobic energy metabolism in cancer may be a key contributor to cancer cachexia.

